# “Double anchor traction method” for large colorectal lesion in colorectal endoscopic submucosal dissection

**DOI:** 10.1055/a-2689-3617

**Published:** 2025-09-04

**Authors:** Keisaku Yamada, Masahiro Tajika, Tsutomu Tanaka, Nobuhito Ito, Akihiro Takagi, Yasumasa Niwa

**Affiliations:** 1 Department of Endoscopy, Aichi Cancer Center Hospital, Nagoya, Japan

In colorectal endoscopic submucosal dissection (ESD), traction devices are effective in reducing procedure time and complications 1 . We developed a novel traction technique using a multi-loop traction device (MLTD; Boston Scientific Co. Ltd., Tokyo, Japan) that enables traction at three locations; we termed this the “anchor traction method” 2 . Although a relatively large lesion can be dissected with a good field of view by traction at three points, the traction points are often insufficient for giant lesions that occupy more than half the circumference of the lumen. We report a case in which two MLTDs were used to dissect such a lesion without complication.

A 78-year-old man presented with a 50-mm Is + IIa lesion at the ascending colon ( Fig. 1 ) and underwent ESD ( Video 1 ). A full circumferential incision was made, and the middle loop of MLTD was attached to the reopenable clip (SureClip; MicroTech, Nanjing, China), and two additional loops of MLTD were then attached to the lesion as previously reported as the anchor traction method. Subsequently, a new MLTD with a clip was delivered and a clip was attached to the loop of the side of preexisting MLTD. By attaching it to the lesion in the same way as the previous MLTD and attaching the loop of the MLTD to the opposite intestinal mucosa, traction was achieved in six locations ( Fig. 2 ). Then, inadequate traction areas were eliminated and the submucosa became clearly visible, allowing ESD with a knife to be performed safely. Pathological analysis revealed that the lesion was a 50×45-mm intramucosal carcinoma with negative margins ( Fig. 3 ).

**Fig. 1:**
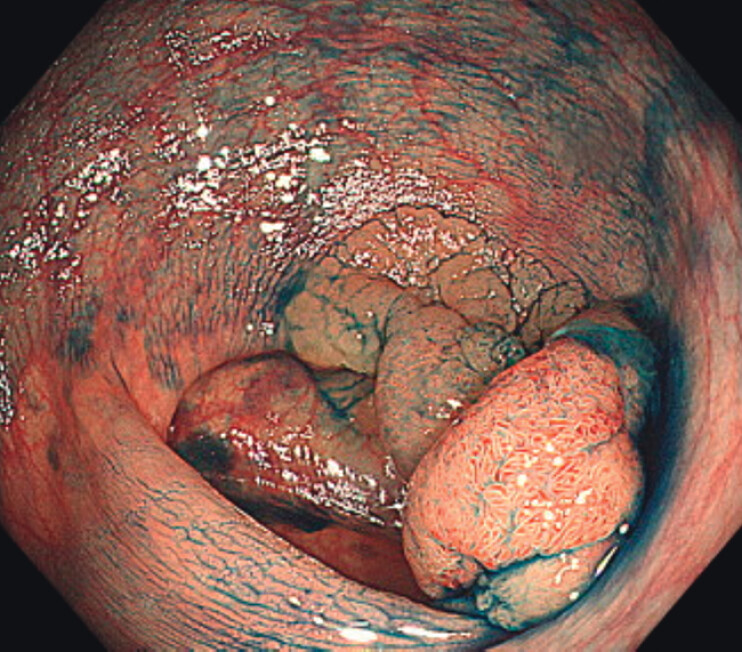
The lesion was a 50-mm 0-Is+ IIa lesion at the ascending colon.

“Double anchor traction method” for large colorectal lesion.Video 1

**Fig. 2:**
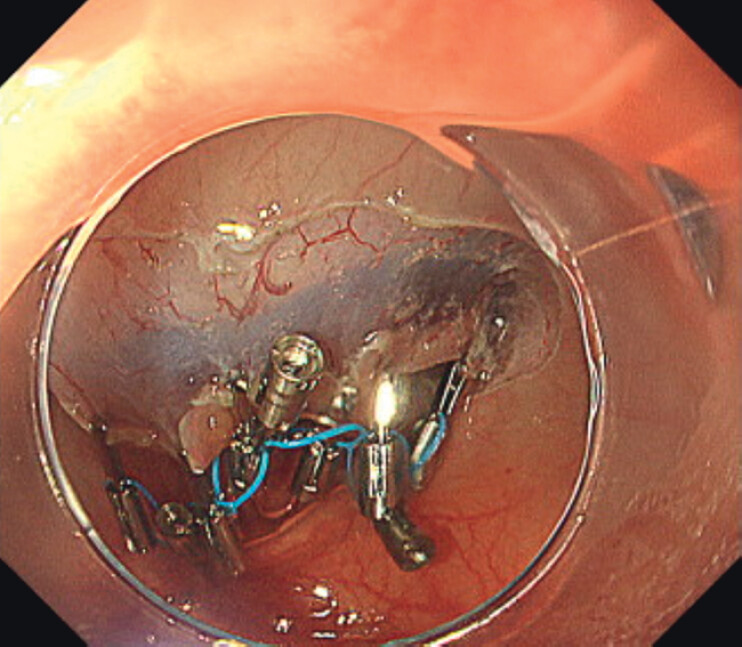
Two multi-loop traction devices were used to perform traction at six points.

**Fig. 3:**
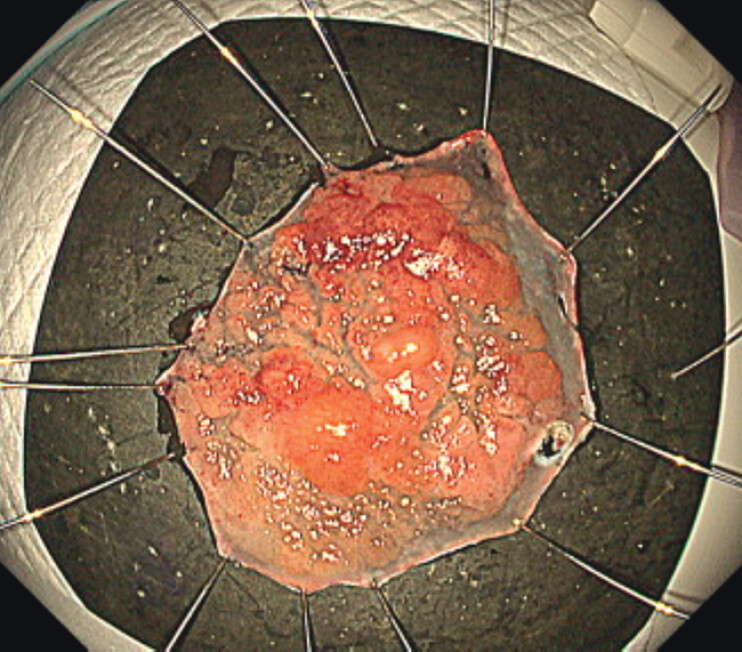
Pathological analysis revealed that the lesion was a 50×45-mm intramucosal carcinoma with negative margins.

This traction method combines two MLTDs to enable traction at multiple locations and is termed the “double anchor traction method.”

Endoscopy_UCTN_Code_CPL_1AJ_2AD_3AD

## Notes

Endoscopy E-Videos https://eref.thieme.de/e-videos
*E-Videos* is an open access online section of the journal *Endoscopy* , reporting on interesting cases and new techniques in gastroenterological endoscopy. All papers include a high-quality video and are published with a Creative Commons CC-BY license. Endoscopy E-Videos qualify for HINARI discounts and waivers and eligibility is automatically checked during the submission process. We grant 100% waivers to articles whose corresponding authors are based in Group A countries and 50% waivers to those who are based in Group B countries as classified by Research4Life (see: https://www.research4life.org/access/eligibility/ ). This section has its own submission website at https://mc.manuscriptcentral.com/e-videos .
